# Metastases and Colon Cancer Tumor Growth Display Divergent Responses to Modulation of Canonical WNT Signaling

**DOI:** 10.1371/journal.pone.0150697

**Published:** 2016-03-03

**Authors:** Chandan Seth, Ariel Ruiz i Altaba

**Affiliations:** Dept. of Genetic Medicine and Development, University of Geneva Medical School, Geneva, Switzerland; University of Colorado, Boulder, UNITED STATES

## Abstract

Human colon cancers commonly harbor loss of function mutations in APC, a repressor of the canonical WNT pathway, thus leading to hyperactive WNT-TCF signaling. Re-establishment of Apc function in mice, engineered to conditionally repress Apc through RNAi, resolve the intestinal tumors formed due to hyperactivated Wnt-Tcf signaling. These and other results have prompted the search for specific WNT pathway antagonists as therapeutics for clinically problematic human colon cancers and associated metastases, which remain largely incurable. This widely accepted view seems at odds with a number of findings using patient-derived material: Canonical TCF targets are repressed, instead of being hyperactivated, in advanced colon cancers, and repression of TCF function does not generally result in tumor regression in xenografts. The results of a number of genetic mouse studies have also suggested that canonical WNT-TCF signaling drives metastases, but direct in vivo tests are lacking, and, surprisingly, TCF repression can enhance directly seeded metastatic growth. Here we have addressed the abilities of enhanced and blocked WNT-TCF signaling to alter tumor growth and distant metastases using xenografts of advanced human colon cancers in mice. We find that endogenous WNT-TCF signaling is mostly anti-metastatic since downregulation of TCF function with dnTCF generally enhances metastatic spread. Consistently, elevating the level of WNT signaling, by increasing the levels of WNT ligands, is not generally pro-metastatic. Our present and previous data reveal a heterogeneous response to modulating WNT-TCF signaling in human cancer cells. Nevertheless, the findings that a fraction of colon cancers tested require WNT-TCF signaling for tumor growth but all respond to repressed signaling by increasing metastases beg for a reevaluation of the goal of blocking WNT-TCF signaling to universally treat colon cancers. Our data suggest that WNT-TCF blockade may be effective in inhibiting tumor growth in only a subset of cases but will generally boost metastases.

## Introduction

There is a large body of evidence that supports the notion that WNT signaling is critical for human colon cancers, which is perhaps best exemplified by two key findings i- the vast majority of colon cancers harbor mutations in the tumor suppressor APC, a key WNT-TCF pathway antagonist [1], and ii- restoration of Apc function in *apc* compromised mice results in the loss of intestinal tumors [2]. These and other results have promoted a search for therapeutic WNT-TCF blockers to universally treat human colon cancers with hyperactive canonical WNT signaling (e.g. [3]). Given that APC is, so far, not druggable, efforts to develop WNT-TCF antagonists have centered on steps upstream (e.g. Porcupine and Tankyrase inhibitors [4–6] or downstream (CBP blockers [7], Ivermectin [8]) from APC function in the ß-CATENIN destruction complex. Independent of the step targeted, many screens or validation assays have used a TCF-luciferase reporter to monitor canonical WNT pathway responses [9].

The lines of evidence summarized above suggest a robust action of WNT-TCF signaling in end-stage colon cancers and can provide a compelling case for the development and use of canonical WNT antagonists. However, a key finding is at odds with this generally accepted view: The expression of WNT-TCF targets is repressed—not enhanced—in advanced colon cancers and metastases as compared with early stage tumors [10], [11].

WNT-TCF signaling is also widely thought to drive metastases (e.g. [12]), promoting the seeding potential of circulating tumor cells and enhancing ligand-mediated signaling in the metastatic niche [13], [14]. It is noteworthy, however, that no direct tests for the accepted ability of enhanced canonical WNT-TCF signaling to promote metastases from human colon cancer cells have been reported. The best evidence for a role of canonical WNT signaling in colon cancer metastases derives from studies with mice [13], [14] or from in vitro analyses (e.g. [15], [16]), and other studies imply a proven role for enhanced WNT signaling in metastases (e.g. [17]). However, assuming in vivo function from in vitro data, or human biology from mouse data, can be problematic as mice and humans are fairly distant species, and in vitro conditions can impose a WNT-dependency to human colon cancer cells [10].

Experimentally, repression of canonical WNT signaling has been attempted by blocking TCF function, which is the last step of canonical WNT signaling. Repression of WNT-TCF signaling with pan dominant-negative TCF (dnTCF4), shown to be effective in vitro on human colon cancer cells (e.g. [18]), is, paradoxically, generally ineffective in repressing tumor growth after grafting advanced human colon cancers; In contrast, it can boost metastatic growth after direct seeding of cancer cells in the lungs [10], [11], [19–21]. These findings raised the possibility that blockade of active WNT-TCF signaling in human colon cancer cells may not be generally therapeutically favorable, unlike what has been claimed (e.g. [2], [18]). Nevertheless, the approaches and model used are different and there could be methodological differences that underlie the divergent results. For instance, directly seeded metastases by tail vein injection of human cancer cells [10] does not fully recapitulate distant metastases. Moreover the accepted idea that WNT ligands promote metastatic growth derived from genetically engineered mice [13], [14], remains to be tested directly using patient-derived colon cancer cells.

Here we have compared gain and blockade of function approaches to directly address the effects of modulating WNT-TCF signaling on human colon cancer xenograft growth in mice and on distant organ metastases in vivo, using both patient-derived primary colon cancer cells and two established cell lines. We find that only a minority of tumor cells tested respond to TCF blockade by diminishing tumor growth, but in contrast all cells tested responded by enhancing distant organ metastases. Moreover, elevated WNT signaling through enhanced ligand levels in cancer cells do not boost tumor growth in any case. Instead, it resulted in a heterogenous metastatic response: enhanced WNT signaling repressed metastatic growth in one case, three cases were statistically unaffected, and it enhanced metastases in another case. Further analysis of the enhancement of metastases in mCC11 cells revealed that this occurred in only one of three immunocompetent mouse strains, arguing that this is not a tumor cell intrinsic response.

Overall, our present and previous data [10] suggest that blockade of WNT-TCF signaling in patients with colon cancer will likely be detrimental in all cases as this will lead to an increase in metastases, independent of any reduction in primary tumor size in a subset of cases. In contrast, the results we report here suggest that restoration of WNT-TCF pathway activity in human colon cancer cells might instead have therapeutic value in a subset of metastatic cancers.

## Materials and Methods

### Human colon cancer samples and cell culture

Primary human colon cancer (CC) samples CC36 (TNM3), CC14 (TNM4) and mCC11 (liver metastasis from CC patient) used for this study were as in references [10], [21], [22], [23]. The human CC HT29 and T84 cell lines, the latter derived from a lung metastasis, were obtained from Cell Line Services. All primary CC cells were used at an early in vitro passage in standard culture conditions (DMEM-F12 with 10%FBS, 5% CO_2_).

### Lentiviral tools and infection of human CCs

Stable introduction of Beta-galactosidase (ßGal)-expressing (*lacZ*^+^) reporter constructs used lentiviral particles at a multiplicity of infection (MOI) of 2. All infected cultures were checked for ßGal expression in vitro by X-Gal staining. Cell culture batches with >90% homogenous expression of ßGal were selected for further infections (MOI = 2) with GFP^+^ lentivectors with or without dnTCF4, Wnt1 or Wnt3a. Batches with >85% GFP^+^ cells were chosen for all experiments.

### Real-time quantitative PCR

3-5d post-infection, infected cells were collected in Trizol and mRNA extracted (Life Technologies). Standard PCR reactions were performed on derived cDNA with the following primers:

Human-specific primers:

TBP F: CCACAGCTCTTCCACTCACA

TBP R: GGATTATATTCGGCGTTTCG

HPRT F: GCCAGACTTTGTTGGATTTG

HPRT R: CTCTCATCTTAGGCTTTGTATTTTG

AXIN2 F: AGTGTGAGGTCCACGGAA AC

AXIN2 R: ACTGCCCACACGATAAGGAG

LGR5 F: GGAGCATTCACTGGCCTTTA

LGR5 R: CTGGACGGGGATTTCTGTTA

ASCL2 F: GCGTTCCGCCTACTCGT

ASCL2 R: GGCTTCCGGGGCTGAG

Mouse-specific primers:

Wnt1 F: GACGGATTCCAAGAGTCTGC

Wnt1 R: ATTGCGAAGATGAACGCTGT

Wnt3a F: GCACCACCGTGGACGACAG

Wnt3a R: CCTCGCTACAGCCACCCCAC

### Tail vein injections in NSG mice: Pure *lacZ*^+^ cell population

10^6^ human CC cells in HBSS were injected in the tail vein of 6-week-old female immunocompromised NSG mice (lacking mature T, B and NK cells) under authorized operating procedures at the animal facility in UNIGE. The animals weighed 20-25g at the start of experiment. The animals were routinely checked for symptoms of discomfort and collected 4 weeks post-inoculation following approved protocols. Lungs were collected in PBS after dissection.

### Subcutaneous xenografts in NSG, Nude and SHO mice: lacZ^+^ cells

5x10^5^ CC cells in HBSS were injected in the subcutaneous layer of 6-week-old female Nude mice (NMRI-Foxn1nu/Foxn1nu, lacking mature T cells) or SHO mice (SHO-PkrdcscidHrhr; lacking mature T cells and B cells) or immune-compromised NSG (NOD.Cg-Prkdcscid Il2rgtm1Wjl/SzJ). Each mouse carried 3 xenografts in the flanks. Nude and NSG mice were bought from Janvier Labs (France) and SHO mice from Charles River (France). All animals were 20-25g at the start of experiment and were subsequently routinely checked for symptoms of discomfort at the time of tumor measurements. Animals were sacrificed at the end of 4 weeks post-grafting and xenografts and organs were collected in PBS after dissection. Xenografts were chopped and stored in Trizol after solubilization and later processed for gene expression analyses by real time quantitative PCR.

### Sub-cutaneous *lacZ*^*+/-*^ xenografts in NSG mice

mCC11 *lacZ*^+^ cells were infected with a lentivector at >90% efficiency. In parallel, mCC11 wt (*lacZ*^-^) cells were infected with GFP^+^ lentivectors expressing Wnt1. mCC11-*lacZ*^+^ were then mixed 1:1 with mCC11-*lacZ*^-^/Wnt1^+^ cells and a total of 5x10^5^ cells were injected in each of three sites per 6-week-old female immunocompromised NSG mice under approved conditions. The animals were routinely checked for symptoms of discomfort daily at the time of tumor growth measurements. All animals were sacrificed at the end of 4 weeks post-grafting and tumors and organs collected in PBS at the end of experiments.

### Beta-galactosidase X-Gal staining

All organs were washed with cold PBS after collection and fixed with 4% PFA at 4°C for 2-12h. Staining was done for 4-8h with X-Gal. Stained samples were then washed with sterile PBS and inspected for visible metastasis under a dissecting microscope. *lacZ*^+^ metastases were counted and photographed.

### Exome sequencing of APC

Exome sequencing of the DNA of the colon cancer primary cells used was performed at the Genomics Facility of the University of Geneva Medical School. Only APC was analyzed in detail for this study. Only known APC mutations leading to loss of function are reported here.

### Animal care

All mice were used under approved protocols from the Office Cantonale de Vétérinaire de Genève (OVC). Animals were monitored several times weekly for well-being under veterinarian guidance. Mice were euthanized at the end of the experiments and before tumors reached the local legal limit through injection of ketazol/xylazine or CO_2_ inhalation.

## Results

### Modulation of canonical WNT signaling in human colon cancer xenografts in mice does not generally alter tumor growth

Primary human colon cancer cells (CC14 and CC36) as well as metastatic colon cancer cells (mCC11) and a metastatic cell line (T84) were stably transduced with GFP^+^ dominant-negative TCF4 (dnTCF), Wnt1, or with control lentivectors. Subcutaneous grafts into immunocompromised NSG mice were chosen since cutaneous colon cancer metastases occur in patients and because no prefect orthotopic grafts are possible in mice: even injection into the rectal wall leads to vessel leakage, given its thinness and the volumes and pressures involved in the injection, leading to systemic dissemination. Moreover, subcutanous grafts are simple and highly reproducible.

**Fig 1:**
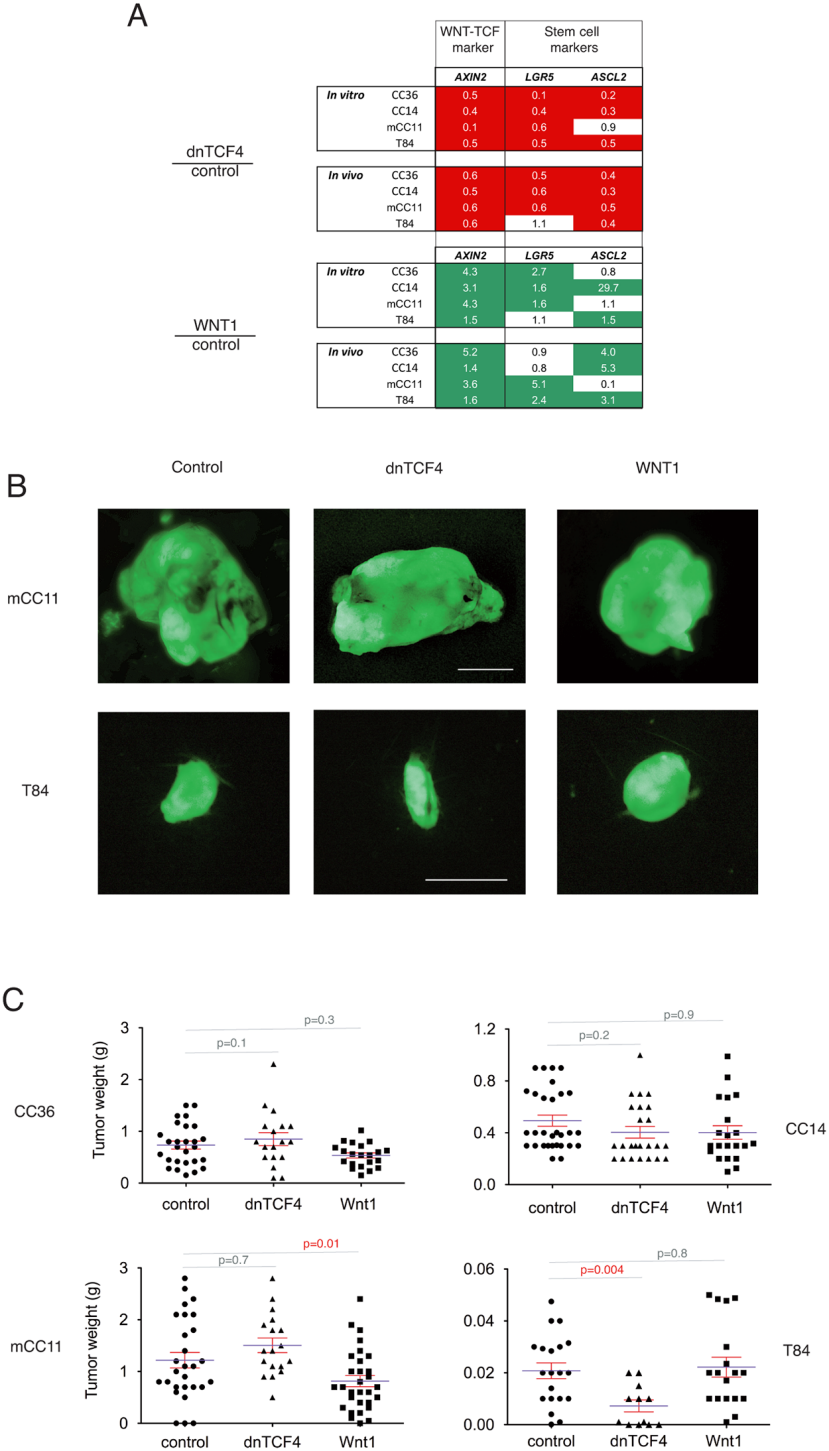
Downregulation or upregulation of canonical WNT-TCF signaling in human colon cancer xenografts does not result in general changes in tumor growth. (A) Quantification of the levels of *AXIN2*, *LGR5* and *ASCL2* by RT-qPCR in vitro and in vivo in xenografts in the four cell types used in this study, with WNT-TCF downregulation via dnTCF4 or upregulation via Wnt1 misexpression. The numbers are the ratios of experimental over control Ct values, after normalization with the housekeeping genes *TBP* and *HPRT*. Note that in all cases the canonical WNT-TCF target and activity reporter *AXIN2* is induced by Wnt1 (green boxes) and repressed by dnTCF4 (red boxes). (B) GFP+ fluorescent images of dissected tumors of mCC11 (top row) and T84 (bottom row) cells. Modulation of WNT-TCF signaling in vivo causes small changes in growing tumors. Scale bars = 0.6cm. (C) Histogram of the quantification of tumor weight after dissection, for each of the four colon cancer cells used. See text for details. p values were determined with two-tailed Student’s t tests.

The levels of expression of the bona fide colon cancer WNT-TCF activity biomarker *AXIN2*, as well as the WNT-regulated stem cell markers *LGR5* and *ASCL2* (e.g. [2]), were determined by RT-qPCR in transduced cells in vitro and in subcutaneous xenografts (Fig 1A). All cells in vitro and in vivo responded to repressed or enhanced WNT-TCF signaling as demonstrated by the endogenous levels of *AXIN2* mRNA; all cells repressed *AXIN2* mRNA levels in response to pathway blockade by dnTCF, and all cells increased *AXIN2* mRNA levels in response to pathway activation by Wnt1 (Fig 1A). *LGR5* and *ASCL2* were similarly regulated albeit with cell type differences (Fig 1A).

Tumor growth was monitored every two days by measuring volume before tumors reached the legal limits. At the end of the experiment, all GFP^+^ tumors were collected at the same time and photographed. In no case were experimental tumors larger than controls (Fig 1B and 1C). Instead, 4/8 T84 xenografts expressing dnTCF (T84^dnTCF^) failed to grow and those that did were within the range of controls. Moreover, there was a minor decrease in tumor weight for mCC11^Wnt1^ as compared with controls (Fig 1B and 1C). These results suggest that up or down modulation of canonical WNT signaling can have context-dependent and divergent effects in vivo. However, in no case did enhanced WNT signaling resulted in larger tumors and blockade of WNT-TCF signaling did not have universal anti-tumor effects.

### Blockade of TCF activity leads to a general increase in distant organ metastases

**Fig 2:**
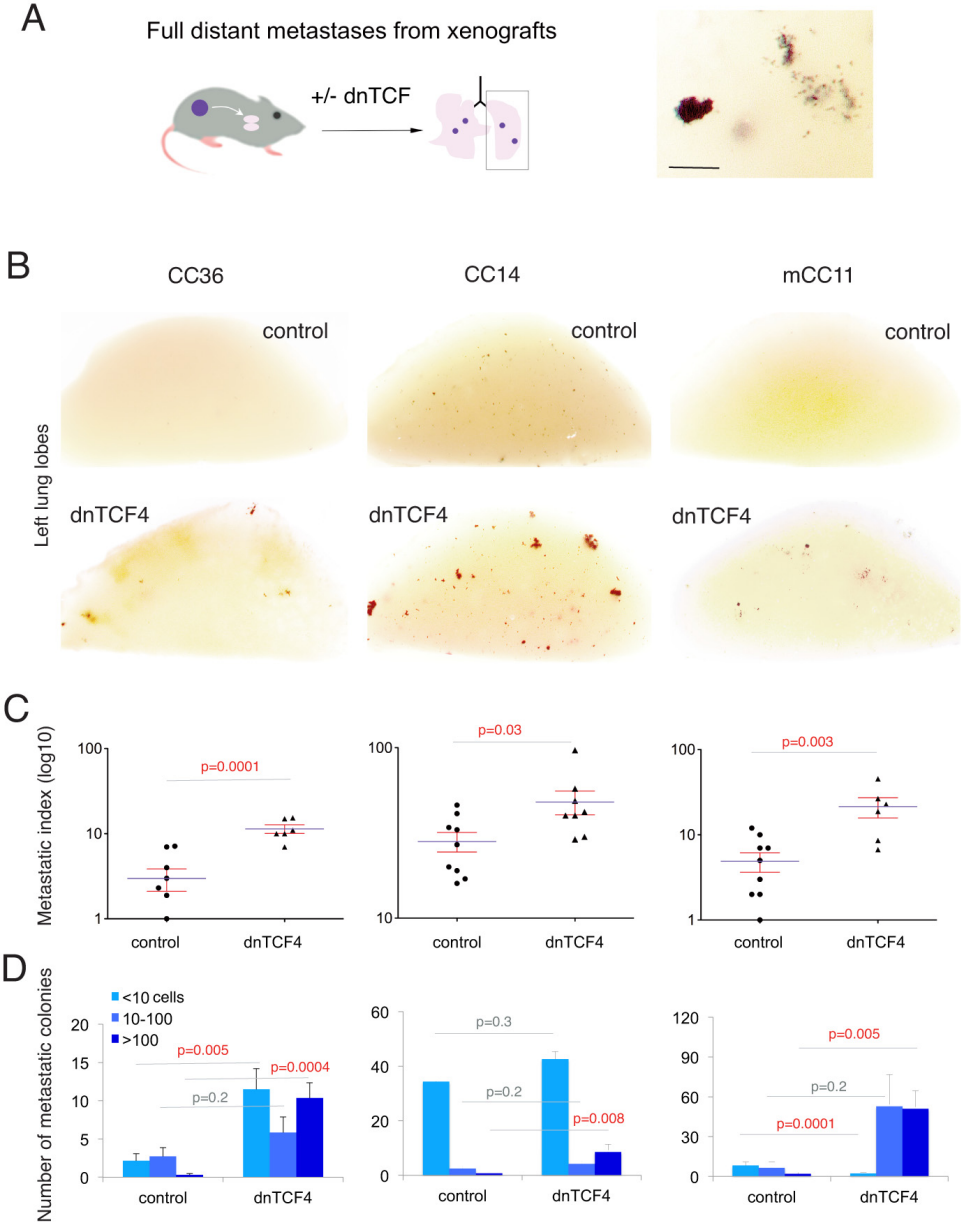
Repression of WNT-TCF signaling in subcutaneous xenografts enhances distant metastases. (A) Diagram of the procedure used to analyze the effect of downregulation of of WNT signaling on distant metastases in vivo. Grafted cells are *lacZ*^+^, which allows the visualization of large colonies and even single metastatic cells in distant organs, notably the lungs (bottom inset). The inset to the right shows a high magnification of *lacZ+* metastases of different sizes after in toto XGAL staining to reveal *lacZ*^+^ metastatic cells (dark blue spots). (B) Representative images of left lung lobes from mice grafted subcutaneously with control cells or those with blocked WNT-TCF signaling through the expression of dnTCF4 as indicated. (C) Quantification of the number of lung metastases per animal shown as the metastatic index (number of metastases over tumor weight) (top row), and tumor weight (bottom row) as indicated in each case. (D) Quantification of the size of lung metastatic colonies as indicated per left lung lobe side. In all figures error bars are s.e.m. and p values derive from unpaired, two-tailed Student’s t tests. Scale bar = 7mm for (A); 25mm for (B).

To clearly track metastatic colonies in distant organs, we grafted cancer cells with integrated *lacZ*-expressing lentivectors to allow the identification of human ßGalactosidase^+^ (ßGal^+^) cells in mouse tissues after the XGal reaction (Fig 2A; [10], [24]). Grafting *lacZ*^+^ CC14^dnTCF^, CC36^dnTCF^ or mCC11^dnTCF^ (Fig 2B) resulted in a net increase in metastases in the lungs of NSG mice, and in their metastatic index (number of *lacZ*^+^ metastatic colonies divided by total tumor weight in grams since each mice carried 2–3 subcutaneous flank xenografts) (Fig 2C). This indicates that down modulation of WNT-TCF activity (by about 50% as suggested by *AXIN2* levels (Fig 1A) enhances the full experimental metastatic phenotype. Given that dnTCF activity prevented the growth of one third (4/12) of T84^dnTCF^ tumors (Fig 1C), a general metastatic response in this cell type was not entirely meaningful to quantify. However, the metastatic index of T84^dnTCF^ mice that developed tumors was similar to that of T84^control^ (not shown).

Quantification of the size of *lacZ*^+^ metastatic colonies in the lungs revealed that there was a consistent increase in the number of large (>100 cells per colony) metastases after repression of WNT-TCF signaling in mice bearing CC36^dnTCF^, CC14^dnTCF^ and mCC11^dnTCF^ xenografts as compared with controls (Fig 2D). Moreover, both CC36^dnTCF^ and mCC11^dnTCF^ tumor-bearing mice also displayed a larger number of micrometastases (<10 cells per colony) over controls (Fig 2D). These results suggest that downregulation of canonical WNT signaling can lead to both an increase in the number of metastatic cells reaching a distant organ as well as an increase in the size of the metastatic colonies.

### Enhancement of WNT ligand levels does not generally increase the number of distant organ metastases

**Fig 3:**
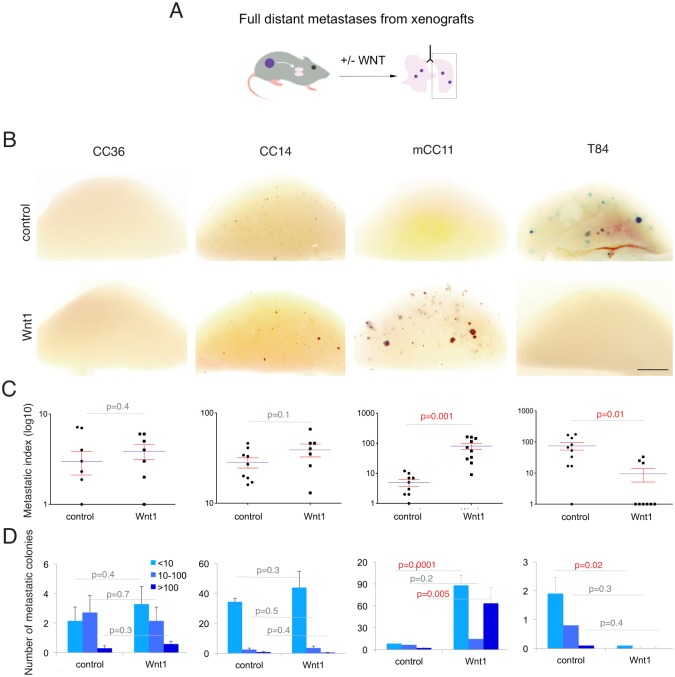
Enhancement of WNT-TCF signaling in subcutaneous xenografts affects distant metastases in a context-dependent manner. (A) Diagram of the procedure used to analyze the effect of upregulation of WNT signaling on distant metastases in vivo. (B) Representative examples of left lung lobes of mice bearing subcutaneous tumors of the different colon cancer cells as indicated, either carrying control (top row) or Wnt1-expressing (bottom row) lentivectors, after X-Gal staining. Scale bar = 25mm. (C) Quantification of the metastatic index in each case per mouse. (D) Quantification of the size of lung metastatic colonies as indicated per left lung lobe side. Note that the controls are the same used for Fig 2 as the Wnt1 and dnTCF experiments were performed simultaneously but are shown separately for clarity.

To test for distant metastases from a primary tumor site to a distant organ, the lungs in this case, control and Wnt1-expressing *lacZ*^+^ cells were grafted into fully immunocompromised NSG mice subcutaneously (Fig 3A). Whereas CC14^Wnt1^, or CC36^Wnt1^ tumors had no significantly altered levels of lung metastases as compared with controls (Fig 3B and 3D), T84^Wnt1^ tumors produced fewer and mCC11^Wnt1^ tumors produce more metastases and had an altered metastatic index and colony size distribution (Fig 3C and 3D). This shows that the metastatic outcome of WNT signaling triggered by ligand action can be context-dependent but that enhanced WNT signaling is not universally pro-metastatic.

### Enhanced WNT signaling modulates the number of directly seeded metastases in a context-dependent manner

**Fig 4:**
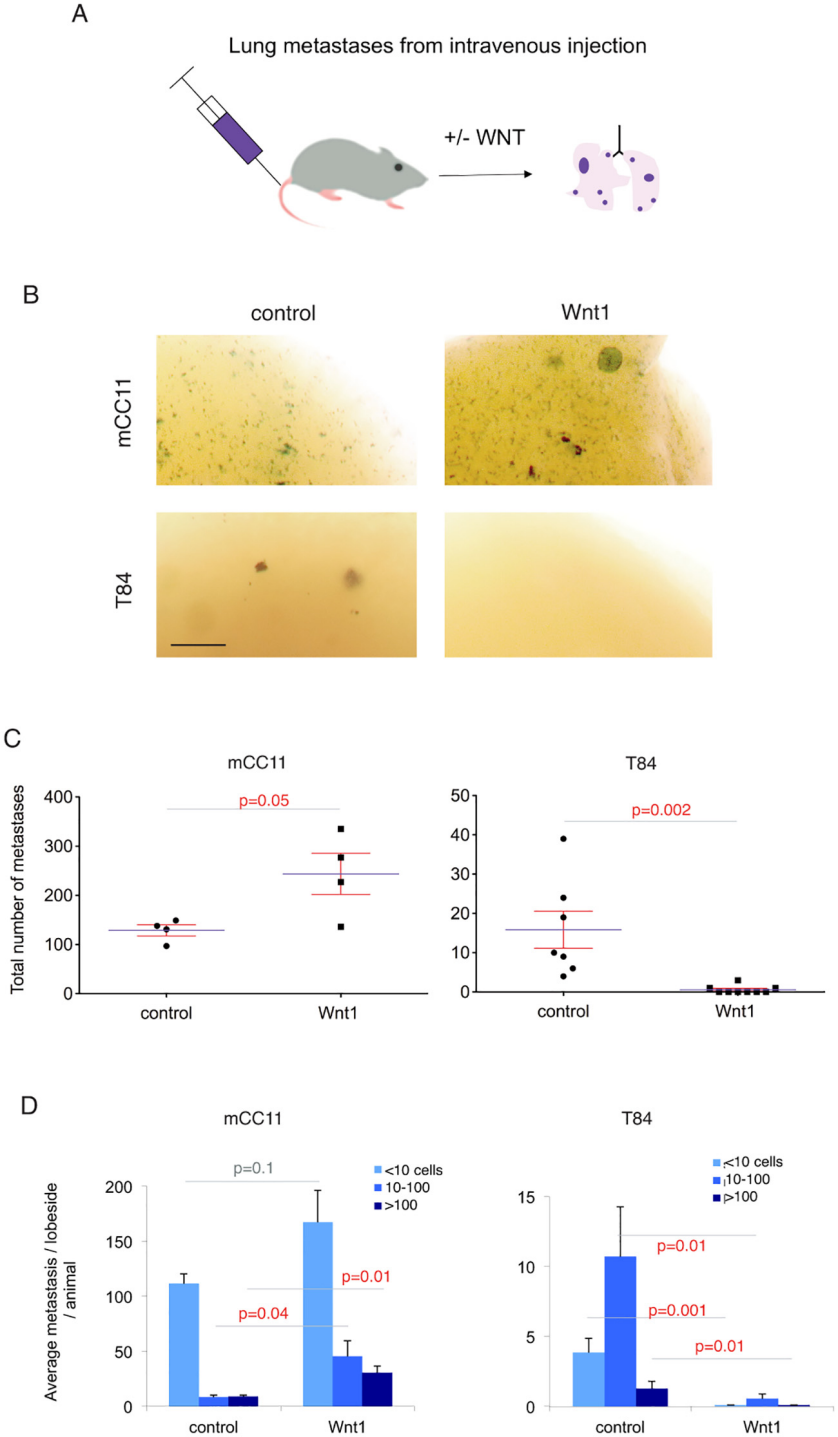
Effects of enhanced Wnt signaling on directly seeded lung metastases. (A) Schematic diagram of the protocol used to test the effects of enhanced WNT signaling on metastatic growth after direct injection into the circulation vial the tail vein. (B-D) Representative examples (B), total quantification (C) and quantification of metastatic colony size (C) of X-Gal stained lung regions after injection of control cells or of cells with enhancement or WNT signaling as indicated. mCC11 metastatic growth is enhanced whereas that of T84 is suppressed (C,D). Note that the total number of metastatic colonies represented in (C) for mCC11 is per left lung lobe, whereas given their scarcity, that for T84 is for the entire lungs. Scale bar = 7mm for (B).

To provide additional support the metastatic phenotype observed with mCC11^Wnt1^ and T84^Wnt1^ cells in the full metastatic assays described above, we directly seeded tumor cells into the lungs, by injecting *lacZ*^+^ mCC11^Wnt1^ or *lacZ*^+^ T84^Wnt1^ cells into the circulation of NSG mice via the tail vein (Fig 4A). mCC11^Wnt1^ cells formed many more and T84^Wnt1^ cells much fewer metastases as compared with controls (Fig 4B and 4C). Quantification of metastatic colonies per size revealed changes in all categories, from single cell micrometastases to large colonies with >100 cells (Fig 4D). These data support the distant metastasis results and argue that WNT signaling can affect metastatic growth, independent of any other effects including on the stroma of the primary tumor. These results also confirm that, paradoxically, enhanced WNT ligand levels are pro-metastatic in one cell type and anti-metastatic in another.

### Lack of correlation of APC status and WNT-TCF modulation phenotype

It could be argued that we have selected cells that may not have mutant APC, thus rendering our results not generally applicable to most human colon cancers. However, we note that T84 harbors *APC* mutations and that exome sequencing of *APC* in CC14 reveals the presence of loss of function mutations (chr5 112173848 G->T, Cosmic id 18566, E^853^->STOP; chr5 112175951 G->A, E^1554^->frameshift).

**Fig 5:**
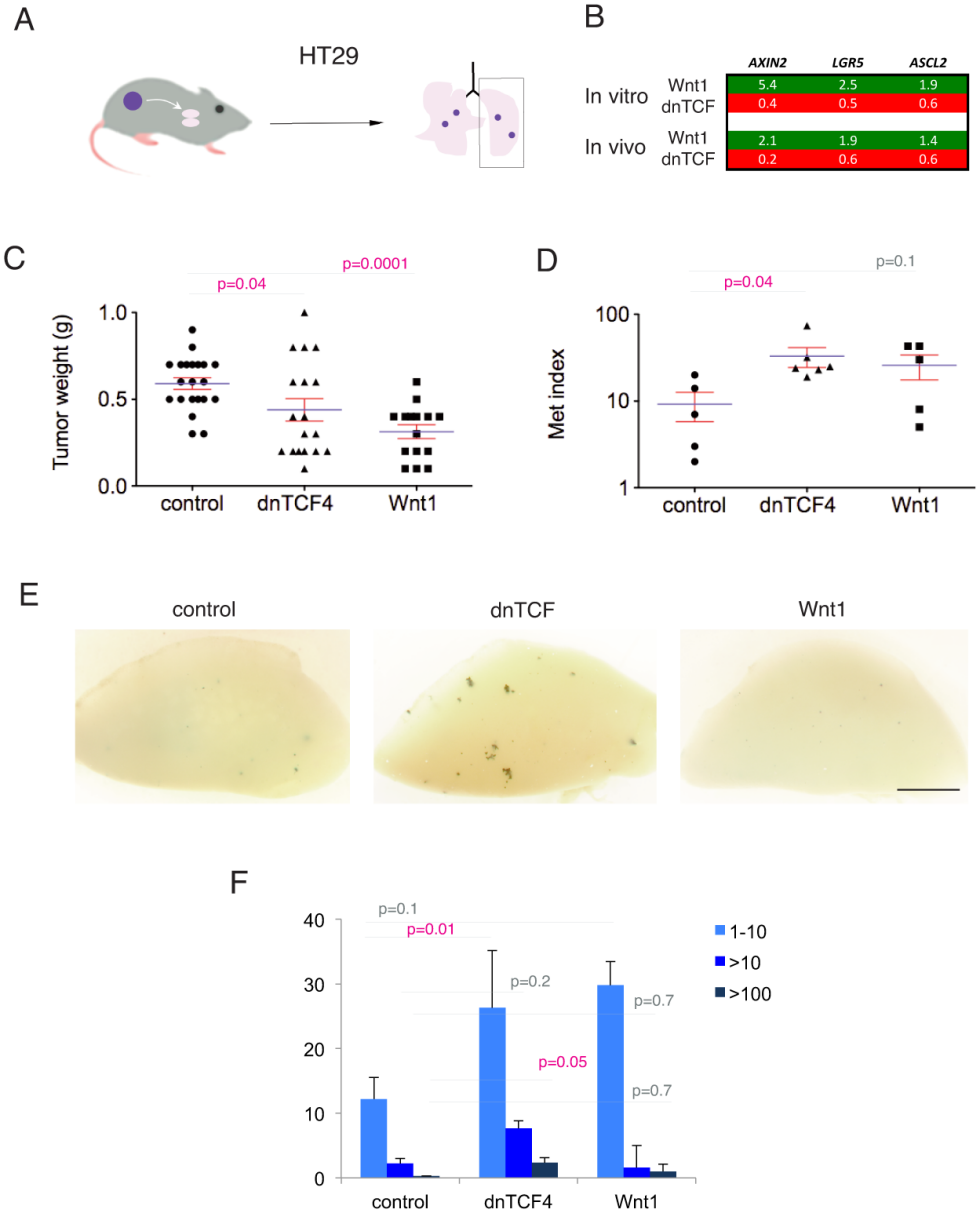
Modulation of WNT signaling in APC mutant HT29 colon cancer cells. (A) Strategy used for subcutaneous to lung metastases. (B) Heat map of normalized mRNA expression levels over controls (and over housekeeping genes) for the highlighted genes in HT29 cells cultured in vitro or in xenografts in vivo. All numbers are ratios over controls. (C,D) Effects of modulation of WNT signaling with dnTCF or Wnt1 expression on subcutaneous tumor growth (C) and metastatic index (Met index) (D). (E) Representative examples of left lung pictures for each case as noted. Metastases are colored dark blue after the XGal reaction due to their cell-intrinsic expression of *lacZ*. (F) Quantification of *lacZ*^+^ metastatic colony size in three categories as shown. Scale bar = 20mm for (E).

To extend these findings we repeated the experiments with dnTCF and Wnt1 using the well-known *APC* mutant colon cancer cell line HT29 (Fig 5A). In these cells Wnt1 and dnTCF4 regulated TCF targets as expected (Fig 5B). Tumors expressing HT29^dnTCF^ were slightly but significantly smaller but this was also the case for HT29^Wnt1^ tumors (Fig 5C), suggesting the lack of a clear tumor growth response to modulation of WNT signaling. Moreover there was no significance difference in number of *lacZ*^+^ metastases from HT29^Wnt1^ tumors as compared with HT29^controls^ (Fig 5D and 5E). In contrast, the increase with HT29^dnTCF^ was significant (Fig 5D and 5E). Analyses of metastatic colony size confirmed increases in micro- and macrometastases from HT29^dnTCF^, but not HT29^Wnt1^, tumors as compared with HT29^controls^ (Fig 5F).

Together, these data support the general notion that cell intrinsic reduction of canonical WNT signaling responses in cancer cells is pro-metastastic, and argue that the status of APC is not simply correlated to the outcome of modulation of WNT-TCF signaling.

### Different canonical WNT ligands enhances the number of distant mCC11 metastases

**Fig 6:**
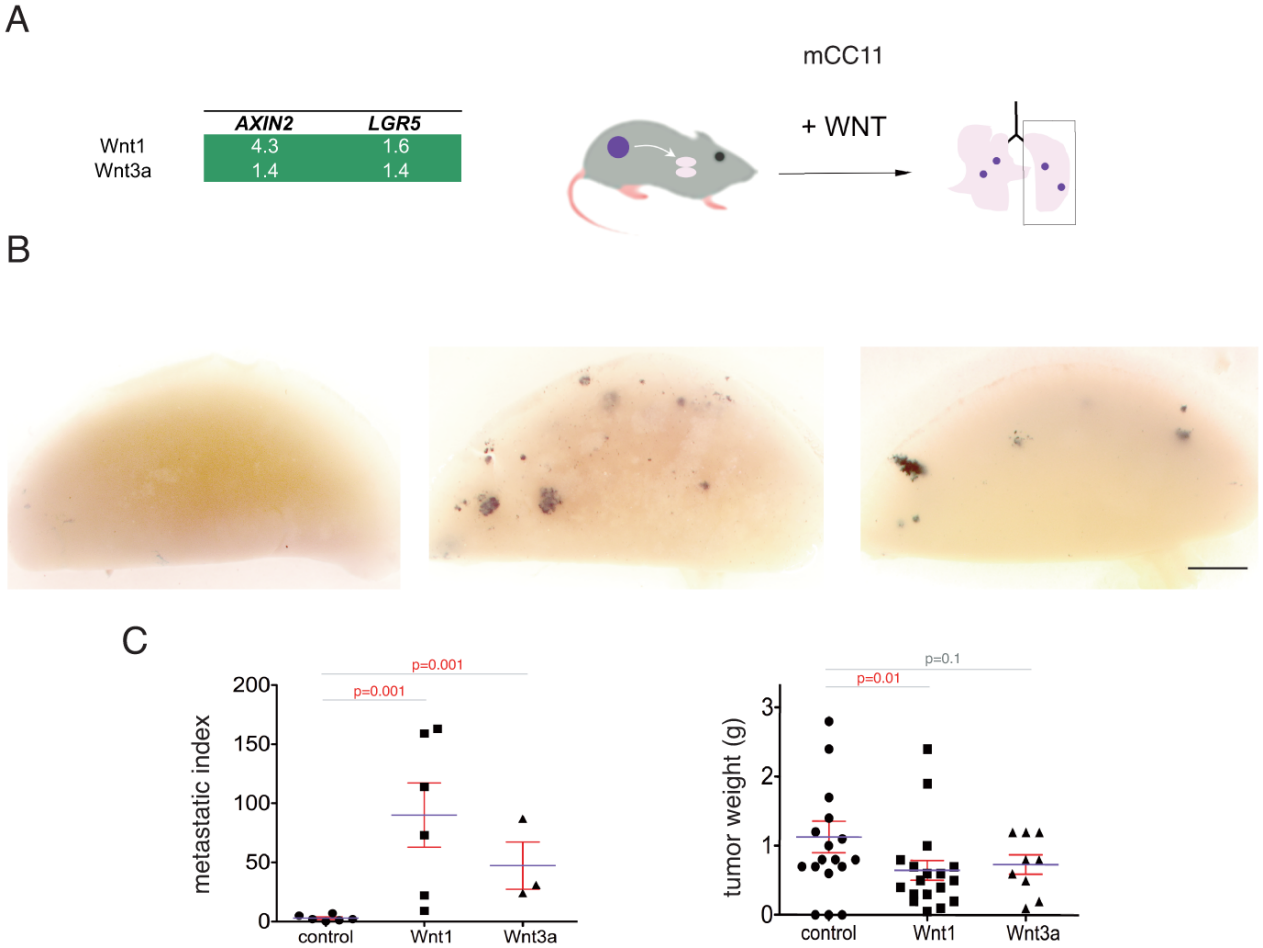
Similar effects of Wnt1 and Wnt3a on the promotion of mCC11 distant metastases. (A) Enhanced Wnt1 and Wnt3a produce similar changes in target gene expression measures by rt-qPCR. (B) Representative examples of X-Gal stained left lung lobes with metastatic colonies as described. (C) Quantification of the metastatic index and the weight for mCC11 wt, mCC11^Wnt1^ and mCC11^Wnt3a^. Both Wnt1 and Wnt3a enhance metastases over controls but only Wnt1 tumors are slightly smaller. Scale bar = 20mm for (B).

To rule out the possibility that the effects of increased metastases by enhanced Wnt1 were due to a specificity of the ligand used, we repeated the full metastatic protocol with mCC11 cells expressing Wnt3a or Wnt1, both of which activate canonical WNT-TCF signaling and, as expected, enhance the levels of the WNT-TCF target *AXIN2* and the WNT-regulated colon stem cell marker *LGR5* over those in control cells (Fig 6A). mCC11^Wnt3a^ *lacZ*^+^ cells produced tumors similar to controls in size but reproduced the enhancement of distant lung metastases from mCC11^Wnt1^ tumors with similar colony size distribution (Fig 6B and 6C and not shown).

### mCC11^Wnt1^ cells promote metastases from sibling non-Wnt1-expressing cells

**Fig 7:**
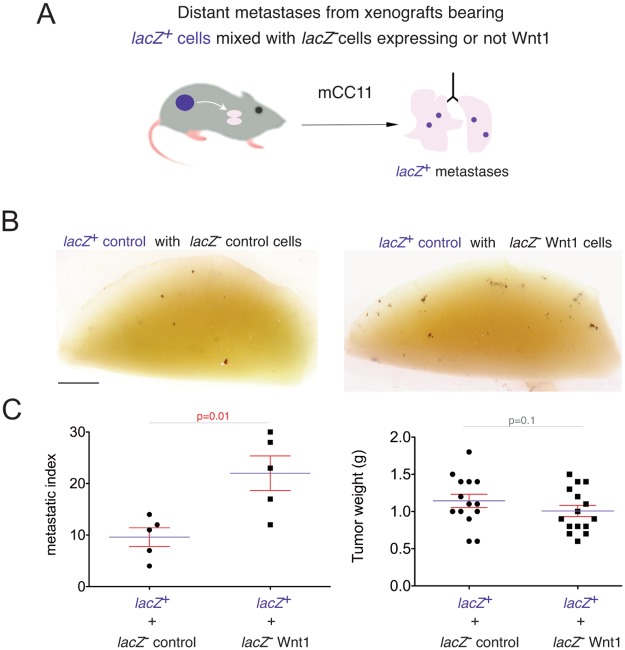
Non-autonomous effect of WNT signaling in mCC11-Wnt1 cells in vivo. (A) Diagram of the protocol used to test the non-autonomous effects of overexpressed Wnt ligands on sibling, non-overexpressing mCC11 cells grafted at the same time. Only *lacZ*^+^;*Wnt1*^-^ cells are tracked after the XGAL reaction. (B) Representative images of X-Gal stained left lung lobes under the described conditions. (C) Quantification of the metastatic index and tumor weight per condition. Scale bar = 25mm for (B).

To ascertain that WNT ligands from mCC11^Wnt1^ cells exert non-cell autonomous signaling in vivo, *lacZ*-negative mCC11^Wnt1^ cells were mixed with *lacZ*+ wt sibling mCC11 cells and co-injected to form subcutaneous tumors (Fig 7A). After tumor growth we tested for the enhanced presence of ßGal^+^ mCC11 wt cells in the lungs. mCC11^Wnt1^ cells significantly promoted the metastatic behavior of sibling mCC11 wt cells (Fig 7B and 7C), indicating that Wnt1 ligands can also act non-autonomously in this context.

### mCC11^Wnt1^ cells do not show increased metastases over controls in NUDE or SHO mice

The results presented above show that canonical WNT signaling is pro-metastatic in mCC11 cells, and that Wnt ligands act both autocrinely and non-autonomously in vivo, suggesting that the primary tumor stroma is unlikely to be the sole target of pro-metastatic WNT signaling. However, we also find that cell-autonomous repression of TCF function with dnTCF leads to a marked increased in distant organ metastases from these cells, as with all other cells tested. We thus hypothesized that blockade of WNT-TCF signaling in mCC11 cells could be intrinsically and generally pro-metastatic (in line with the results in the other tested cells), but that enhanced WNT signaling may boost metastases from mCC11 cells through a non-cell autonomous, mCC11-specific mechanism.

**Fig 8:**
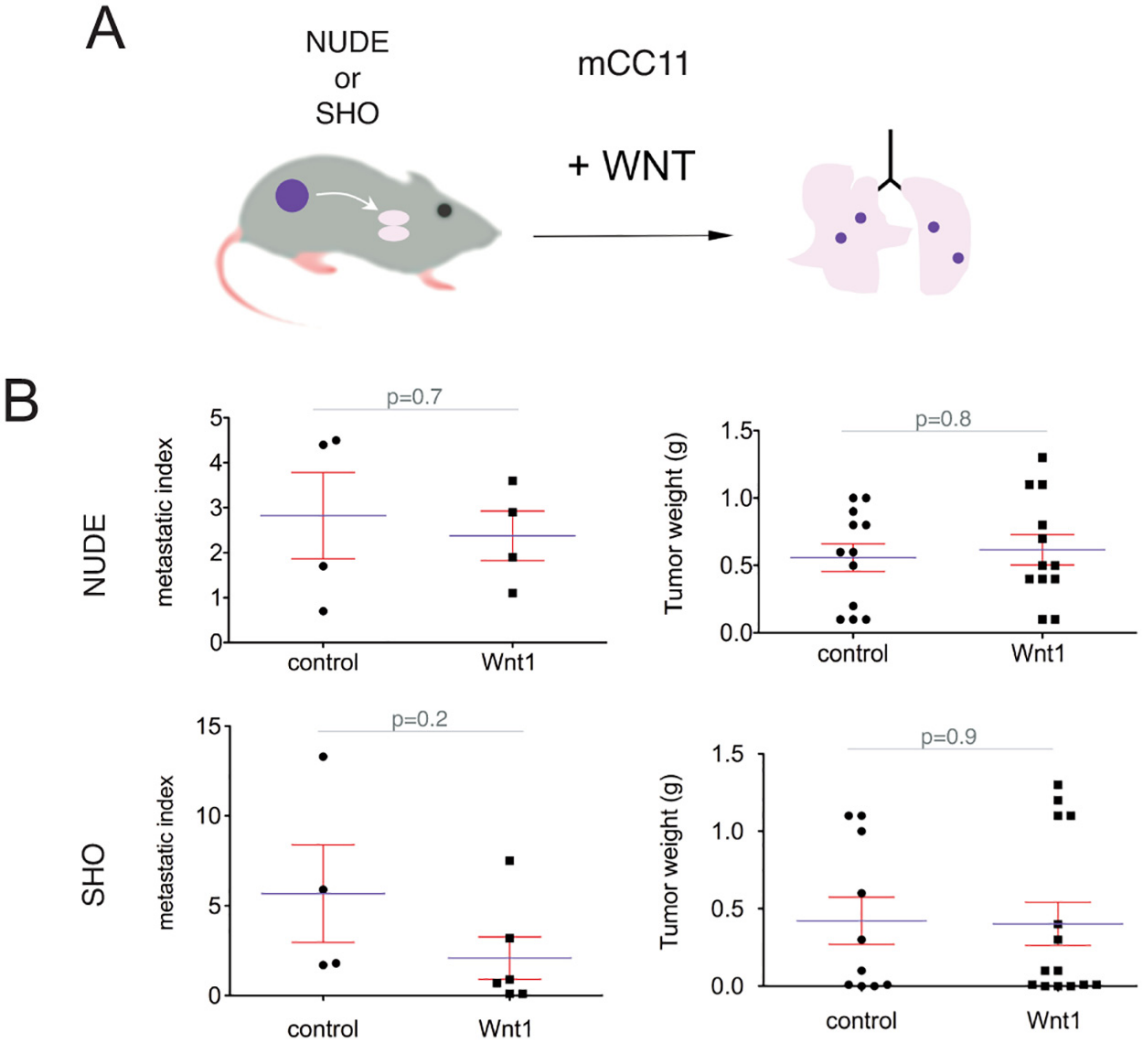
Mouse strain-dependence of enhanced metastases by WNT signaling in mCC11 cells. (A) Diagram of the procedure used to engraft mCC11 cells subcutaneously and the analyses of resulting distant lung metastases. (B) Quantification of tumor weights and metastatic indexes showing lack on promotion of metastases by mCC11^Wnt1^ cells grafted onto NUDE or SHO mouse hosts.

To test if the enhanced metastatic phenotype of mCC11^Wnt1^ depended on the milieu, we radically altered the environment of the grafted tumor cells by changing the strain of the mouse hosts (Fig 8A). mCC11^Wnt1^ subcutaneous tumors grew as controls did in NUDE or SHO mice (Fig 8B and 8C) but, in contrast with the potent metastatic phenotype of mCC11^Wnt1^ cells in NSG mice described above, mCC11^Wnt1^ tumors did not show an increased number of metastases as compared with controls in grafted NUDE or SHO mice (Fig 8B and 8C). These results suggest that mCC11 cells do not display a cell intrinsic metastatic enhancement in response to increased WNT signaling.

## Discussion

Ninety three percent of human colon cancers harbor hyperactivation of the canonical WNT pathway, most often through loss of function of APC (http://cancergenome.nih.gov/). This, together with the results with genetic mouse models, has provided support for a functional role of hyperactive Wnt-Tcf signaling in the initiation and development of intestinal tumors with cells in culture in vitro and in mouse models in vivo (e.g. [2], [18], [25], [26]). Several lines of evidence derived also from work with transgenic mice have shown suggested that the enhancement of Wnt activity, in the metastatic niche through the action of Periostin [13] or by promoting the migratory behavior of circulating tumor cells [14], drives metastases in different cancer types. These and other data have been taken to suggest that high canonical WNT signaling in different human cancers, including those of the colon, is essential for tumor growth and metastases in patients, arguing for the development of therapeutic WNT-TCF pathway antagonists (e.g. [3]). Whereas local tumors such as those in the intestine can be successfully removed by surgery, metastases remain incurable and represent the key target for anti-cancer therapies.

Analyses of patient tumor samples, however, are at odds with the general view presented above: surprisingly, TCF targets are downregulated in advanced colon cancers and metastases as compared with early tumors [10], [11]. Moreover, results with human primary cancer xenografts in mice suggest an alternative to the widely accepted view that canonical WNT signaling is essential for advanced intestinal tumor growth and metastases: repression of WNT-TCF signaling with dnTCF4, the same tool used to show the in vitro WNT dependency of human colon cancer cells [18], is generally ineffective to decrease tumor burden in xenografts in mice. Only 1/5 (DLD1) colon cancer cell types tested showed reduction in tumor volume [10]. In contrast, 4/4 cell types tested that formed tumors with compromised TCF activity yielded increased metastatic growth after injection of cells into the bloodstream and direct lung seeding [10]. The idea that WNT signaling might be anti-metastatic also derived from the result of an unbiased, in vivo, genome-wide shRNA screen that revealed two positive WNT modulators as colon cancer metastatic suppressors [21].

A potential problem with of the metastatic assays with dnTCF-expressing cells described above is that cells were directly seeded into the lungs. Here we have obtained consistent results testing for the formation of distant metastases from subcutaneous xenografts using three human colon cancer patient-derived heterogeneous (not cloned) cell types and one colon cancer cell line (HT29). Moreover, the present data also indicate that enhanced WNT signaling does not promote xenograft growth or metastases, a result that is against all expectations from the still widely accepted notion that canonical WNT signaling is essential for advanced colon cancers and metastases.

Another important outcome from our present work is that human colon cancer cells show divergent responses to altered WNT signaling in an in vivo experimental setting. We find that all colon cancer cells tested respond to modified WNT signaling molecularly, albeit in a context-dependent target-specific manner, but this does not enhance their in vivo metastatic potential in 4/5 cases in NSG mice. Enhanced WNT signaling does enhance metastases from mCC11 tumors but we show this is dependent on the host strain as it is not observed in SHO or NUDE mice. It is interesting to note that the last two have NK cells and that these have been implicated in suppressing metastases [27], [28], although the mechanism by which enhanced WNT signaling boosts metastases from mCC11 subcutaneous tumors in an NSG context remains to be understood. In contrast, the ability of high WNT signaling to suppress directly seeded T84 metastatic growth might suggest that pathway enhancement could have therapeutic relevance in a small subset of cases.

It could be argued that the downregulation of TCF function we induce is not enough to repress all relevant WNT signaling in all colon cancer cells tested. In this and other studies downregulation of TCF function has been afforded by dnTCF4 (e.g. [10], [18]) and the downregulation we report affects the growth of T84 and HT29 tumors (albeit very slightly in the latter case; this work) and similar levels are effective in repressing DLD1 colon cancer cell xenograft growth [10]. Thus, whereas we cannot rule out the possibility that much higher repression would be needed to inhibit tumor growth of all colon cancer xenografts, even partial inhibition should not be detrimental in order for this approach to be valuable. In this sense we show here that partial downregulation of TCF function leads to enhanced metastases to a distant organ in all cases where tumors grow. This result, together with the effects on directly seeded cells in the lungs, argue that blockade of WNT-TCF signaling in patient tumors may engender unwanted effects due to increased metastases.

Generally, our data should signal caution regarding the goal to universally block canonical WNT signaling for the general treatment of colon cancers since pharmacological blockage is unlikely to be complete in all tumor cells of any patient and such treatment may phenocopy our experimental setting. Given the divergent responses to modified canonical WNT signaling by human colon cancer cells we find here, finding functional classifiers, in addition to genomic profiling [29], to stratify patients whose tumors may respond in one way or another to altered WNT-TCF signaling is a priority.
