# Correction: Metastases and Colon Cancer Tumor Growth Display Divergent Responses to Modulation of Canonical WNT Signaling

**DOI:** 10.1371/journal.pone.0160904

**Published:** 2016-08-04

**Authors:** Chandan Seth, Ariel Ruiz i Altaba

There is an error in the last sentence of the penultimate paragraph of the Introduction. The correct sentence is: Further analysis of the enhancement of metastases in mCC11 cells revealed that this occurred in only one of three immunocompromised mouse strains, arguing that this is not a tumor cell intrinsic response.
